# The British Dental Journal

**Published:** 1899-03

**Authors:** 


### The British Dental Journal.

This new journal was established one year ago, and is published monthly in London. It is filled with good things and will be helpful to every dentist who may read its contents.

